# A Comparison between ^**18**^F-FDG PET/CT Imaging and Biological and Radiological Findings in Restaging of Hepatoblastoma Patients

**DOI:** 10.1155/2013/709037

**Published:** 2013-08-26

**Authors:** Angelina Cistaro, Giorgio Treglia, Manuela Pagano, Piercarlo Fania, Valentina Bova, Maria Eleonora Basso, Franca Fagioli, Umberto Ficola, Natale Quartuccio

**Affiliations:** ^1^Positron Emission Tomography Centre, IRMET S.p.A., Euromedic Inc., 10136 Turin, Italy; ^2^Co-ordinator of PET Pediatric AIMN InterGroup, 10136 Turin, Italy; ^3^Associate researcher of Institute of Cognitive Sciences and Technologies, National Research Council, 00185 Rome, Italy; ^4^Department of Nuclear Medicine and PET/CT Centre, Oncology Institute of Southern Switzerland, 6500 Bellinzona, Switzerland; ^5^Pediatric Unit, Hospital Cirie' ASL To4, 10073 Cirie' (TO), Italy; ^6^Department of Radiological Sciences, HSR Giglio, 90015 Cefalù (PA), Italy; ^7^Paediatric Oncohematologic Unit, Department of Paediatric Oncology, Regina Margherita Children's Hospital, 10126 Turin, Italy; ^8^Department of Haematology, Oncology, Immunology and Infectious Diseases, Regina Margherita Children's Hospital, 10126 Turin, Italy; ^9^Nuclear Medicine Unit, La Maddalena Hospital, 90146 Palermo, Italy; ^10^Nuclear Medicine Unit, Department of Biomedical Sciences and of Morphologic and Functional Images, University of Messina, 98125 Messina, Italy

## Abstract

*Background*. In this study we retrospectively evaluated if ^18^F-FDG-PET/CT provided incremental diagnostic information over CI in a group of hepatoblastoma patients performing restaging. *Procedure*. Nine patients (mean age: 5.9 years; range: 3.1–12 years) surgically treated for hepatoblastoma were followed up by clinical examination, serum *α*-FP monitoring, and US. CI (CT or MRI) and PET/CT were performed in case of suspicion of relapse. Fine-needle aspiration biopsies (FNAB) were carried out for final confirmation if the results of CI, PET/CT, and/or *α*-FP levels were suggestive of relapse. PET/CT and CI findings were analyzed for comparison purposes, using FNAB as reference standard. *Results*. *α*-FP level was suggestive of disease recurrence in 8/9 patients. Biopsy was performed in 8/9 cases. CI and PET/CT resulted to be concordant in 5/9 patients (CI identified recurrence of disease, but ^18^F-FDG-PET/CT provided a better definition of disease extent); in 4/9 cases, CI diagnostic information resulted in negative findings, whereas PET/CT correctly detected recurrence of disease. ^18^F-FDG-PET/CT showed an agreement of 100% (8/8) with FNAB results. *Conclusions*. ^18^F-FDG-PET/CT scan seems to better assess HB patients with respect to CI and may provide incremental diagnostic value in the restaging of this group of patients.

## 1. Introduction

Primary liver tumors in pediatric patients represent a heterogeneous group of neoplasms with about 60% of malignant forms. Hepatoblastoma (HB) is the most common primary malignant hepatic tumor in childhood [1, 2]. Elevation of serum alpha-fetoprotein (*α*-FP) is seen in almost 90% of cases [3, 4]. At present, complete resection is possible in more than 50% of cases, and preoperative chemotherapy has been successfully used in converting unresectable to resectable tumors [5–7]. Followup by means of correlation of serial *α*-FP measurements and abdominal sonography (US), performed every 3 months for the first 2 year, may be helpful in detecting relapse [8]. 

In the follow-up period, a rising level of serum *α*-FP is generally associated with tumor recurrence; however, most protocols require additional imaging follow up, since a neoplastic mass needs to be localized by US or CT [9–11]. However, a negative imaging investigation does not exclude the presence of recurrent disease [12] and only biopsy can confirm it [3, 8, 13]. To our knowledge, there are a few reports in the literature investigating the role of 2-deoxy-2-[(18)F]fluoro-D-glucose positron emission tomography/computed tomography (^18^F-FDG-PET/CT) in children with hepatoblastoma. In this retrospective study, we evaluated if ^18^F-FDG-PET/CT provided incremental diagnostic information over conventional imaging (CI) in a group of 9 hepatoblastoma patients undergoing restaging/followup after surgery.

## 2. Methods

Nine patients (mean age: 5.9 years; range: 3.1–12 years) in followup with a history of hepatoblastoma were retrospectively evaluated. All patients were staged using the SIOPEL staging system [18] and received chemotherapy according to the SIOPEL protocol [19]: 4 cycles of PLADO (cisplatin plus doxorubicin) and 2 cycles after transplantation (entire liver in 2/9 patients; left lateral segment from cadaveric donor in 4/9) or surgery (in 3/9 cases). At the end of treatment, all patients were monitored by serial US and measurements of *α*-FP level, every three months for a median follow-up of 24 months. In all patients, further investigations, including CI (CT or MRI) and PET/CT, were requested within 1 week in case of increase of *α*-FP serum level or clinical suspicion of relapse. ^18^F-FDG-PET/CT was performed in case of biochemical relapse or findings on CI consistent with recurrence to better evaluate the possible presence of additional lesions. ^18^F-FDG-PET/CT was also asked by the pediatric oncologist, after negative or inconclusive CI and *α*-FP results, if the suspicion of recurrence was still high on the basis of the clinical examination. Maximum time interval between CI (CT or MRI) and PET/CT was 1 month. Fine-needle aspiration biopsy (FNAB) was carried out for final confirmation if the results of imaging studies and/or *α*-FP levels were suggestive of relapse and used as reference standard to compare the imaging results (PET/CT versus CI). 

### 2.1. PET/CT Technique

The whole-body PET/CT scan was done under fasting condition, 60 minutes after i.v. injection of a fixed dose of 114 MBq using integrated PET/CT device (Discovery ST-E, General Electric Medical Systems, Milwaukee, WI, USA), scanning from the skull base to the mid thigh, with 5-6 fields of view (15 cm). Firstly, low-dose protocol CT was performed with the purpose of attenuation correction and anatomical localization of PET signal. CT images were acquired on matrix of 512  ×  512 pixels (pixel size  =  1 mm), with a tube voltage of 80 kVp, pitch of helical of 3.75 : 1. Maximum CT tube current was 20 mAs, varying on the basis of patient weight. No intravenous contrast agent was injected. After CT, PET data were acquired in 3D mode on a matrix of 128  ×  128 pixels.

### 2.2. Interpretation of PET/CT Imaging

PET/CT studies were interpreted by means of visual and semiquantitative analysis by means of Xeleris software (General Electric Medical Systems) on the reconstructed attenuation corrected PET and CT images in transaxial, coronal, and sagittal views by two experienced nuclear medicine physicians. Semiquantitative measurements of the tracer uptake using standardized uptake value (SUVmax) were obtained by measuring the ratio of the decay-corrected tracer uptake per gram of tumor to the injected dose, normalized for body weight. The SUVmax values were calculated in the largest tumor deposits to minimize partial volume effects, using the average pixel value within the region of interest. Furthermore, we correlated ^18^F-FDG-PET/CT with biological and radiological results and verified the possible presence of additional findings.

## 3. Results

On the basis of FNAB and followup, there were 8 hepatoblastoma relapses and 1 disease-free patient. *α*-FP serum levels were suggestive of recurrence of disease in 8 out of 9 patients at postsurgical followup. Biopsy was performed in 7/8 patients with increased *α*-FP level revealing positivity in 6/7 cases (results are summarized in Table 1). 

A patient (no. 5, embryonal histotype) showed only mildly raised *α*-FP level but negative CI and PET/CT imaging. As a consequence FNAB was omitted, and the patient did not show recurrence at the followup. In patient no. 2, neither *α*-FP level measurement nor CI was valuable in detecting recurrence, which was depicted only by PET/CT (suggested by the pediatric oncologist due to clinical suspicion) and confirmed by biopsy.

CI and PET/CT resulted to be concordant in 5/9 patients; however, in 4 of them, PET/CT provided a better definition of disease extension; in one case both CI and PET/CT correctly classified the patient as negative. In 4 cases (no. 2, no. 6, no. 7, and no. 9) with negative findings on CI, PET/CT correctly detected recurrence of disease. 

^18^F-FDG-PET/CT showed an agreement of 100% (8/8) with FNAB results, while CI results were concordant with FNAB results in 4/8 patients (case nos. 1, 3, 4, and 8). Overall, ^18^F-FDG-PET/CT provided a better diagnostic performance with respect to CI in the entire group of patients and a more accurate definition of the extent of disease in patients presenting recurrence. In one patient, CI correctly detected hepatic recurrence but missed additional abdominal findings, which were showed on ^18^F-FDG-PET/CT scan. In another case, CI correctly restaged hepatoblastoma (hepatic recurrence and pulmonary metastases), whereas ^18^F-FDG-PET/CT showed additional bilateral lung lesions. In patient no. 4, CI correctly detected local recurrence in the liver, but PET/CT depicted a higher number of lesions, while in patient no. 8, CI was suggestive of recurrence, but PET/CT documented an additional perisplenic lesion. 

## 4. Discussion 

US is a noninvasive imaging method for the followup of young patients affected by hepatoblastoma. However, US provides less anatomic detail than CT and is less accurate in the detection of hepatic tumors [20]. In addition, US may not be as sensitive in the evaluation of the postoperative bed due to the presence of either omental flap or gas-filled loops of bowel [1]. CT is currently the modality of choice to diagnose, evaluate preoperatively, and follow up liver tumors in children [21]. Postoperative changes in the liver are common and often do not reflect recurrent or residual tumor: after liver resection, large mattress-type sutures are usually placed into the remaining liver at the resection margin; these can cause necrosis of adjacent hepatic tissue, which may result in postoperative CT findings of low attenuation and calcification. Moreover, changes in the liver remote from resection margins may be due to intraoperative trauma. Changes in attenuation on CT scan are found also after chemotherapy and do not correlate well with tumor necrosis on pathologic analysis [9]. Actually, MRI, which usually requires sedation in children, is reserved mostly to document a recurrent tumor and for instances of allergy to iodine [1].

^18^F-FDG PET/CT is a recent nuclear medicine technique that merges functional with anatomic imaging to optimize disease assessment and evaluation of response to therapy. ^18^F-FDG uptake in tumors is proportional to the metabolic rate of viable tumor cells, which have an increased demand for glucose than normal tissue. Observations with electron microscopy of ultrastructural characteristics of hepatoblastoma cells have demonstrated prominent glycogen granules in the cytoplasm [22, 23]. The presence of copious glycogen in hepatoblastoma cells suggests active accumulation of glucose and its transformation and accumulation in glycogen granules [24]. This observation can explain the uptake of ^18^F-FDG. ^18^F-FDG PET/CT has no established role in initial diagnosis of hepatoblastoma [25] but is helpful in detecting early recurrence [26], and few studies have evaluated its role in followup and restaging of patients after chemotherapy and surgery ([12, 14–17], Table 2). 

Figarola et al. [12] documented a case in which a suspicion of hepatoblastoma recurrence after completion of chemotherapy was confirmed by ^18^F-FDG-PET/CT which correctly identified three pathological nodules in the liver, whereas CT alone missed the anatomical detection. Philip and colleagues, instead, reported in their article about three hepatoblastoma patients; in one case, rising *α*-FP levels and the failure to detect recurrent disease by CI led to the use of FDG-PET. The PET study confirmed local recurrence, leading to prompt surgical intervention that may have been delayed otherwise [16]. Another manuscript describes the use of FDG-PET in a series of 7 children (11 scans) with primary hepatic malignancies (5 patients with hepatoblastoma and 2 patients with hepatic embryonal rhabdomyosarcoma), together with other imaging (CT and MRI), serum tumor markers, and tumor pathology. Abnormal uptake was demonstrated in 6 of 7 patients. In one patient, intense uptake was due to necrotizing granulomas [17]. Another important study is that of Wong et al. [15], who suggest caution in the interpretation of PET studies due to the risk of false positive results. In this retrospective article including sixteen children, three posttreatment patients had PET results suggestive of tumor recurrence. One of these patients had normal *α*-FP level and suspected recurrence in the caudate lobe. Radiologic-guided biopsy was performed 3 times, and there was no evidence of tumor. The other 2 patients underwent further liver resections because of mildly raised *α*-FP levels. The histology results showed regenerative liver tissue only in both patients with no hepatoblastoma recurrence [15]. 

**Figure 1:**
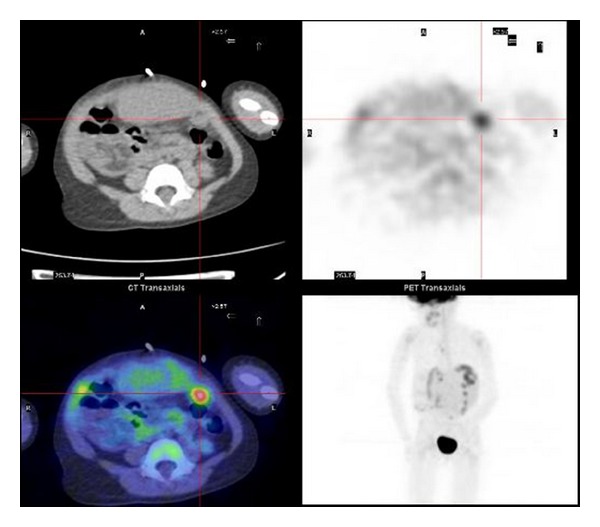
Peritoneal recurrence of hepatoblastoma. ^18^F-FDG-PET/CT transverse images show a peritoneal nodular mass with abnormal FDG uptake suspicious for recurrent hepatoblastoma. The biopsy confirmed the diagnosis.

Our series is one of the biggest in the literature and confirms the findings of previous papers. One of the most important points of this paper was the correlation of PET/CT and CI results with biopsy. PET/CT showed a higher detection rate than CI (100% versus 44%), and we proved that the technique could provide an additional means of evaluating hepatoblastoma patients after surgical approach (liver transplantation or hepatectomy) in the detecting sites of recurrence (Figure 1).

In fact, PET imaging demonstrated more extensive disease involvement than that revealed by other imaging modalities in 7/9 patients. CI failed in detecting one hepatic recurrence and a considerable number of extrahepatic lesions. Biomarker measurements registered a false positive case (mild increase *α*-FP level with no disease recurrence detection at both PET/CT and CI) and a false negative result. These results can be explained since *α*-FP is not always a reliable marker in infants with hepatoblastoma, due to the physiologically elevated levels of *α*-FP in this age group [27] or due to the lack of *α*-FP production in undifferentiated tumors [28]. Despite this aspect, *α*-FP, being a noninvasive and high-sensitive method in detecting recurrence, is considered the gold standard in this setting [29] and allows decreasing frequency of imaging surveillance examinations and radiation exposure. Furthermore, in the routine clinical practice many institutions use to perform at least an additional measurement of *α*-FP level to minimize risk of false positive.

Overall, our results enforce the role of ^18^F-FDG PET/CT in the restaging of hepatoblastoma patients after chemotherapy and surgery. From our point of view, although ^18^F-FDG PET/CT has no definite role in the staging workup of hepatoblastoma [24], it could provide incremental diagnostic value in the initial evaluation of patients affected by hepatoblastoma, helping to detect additional metastatic sites at diagnosis. However, since experience is so far limited in the literature, multicentre and prospective studies are warranted to validate the role of ^18^F-FDG PET/CT in this latter setting. Potential limits of ^18^F-FDG-PET/CT are lesion dimensions, because of PET maximal spatial resolution of 4 mm. In addition, possibility of false positive and false negative should be taken into account. The minimal mitotic activity of the pure fetal subtype may demonstrate lower FDG avidity than other more unfavorable hepatoblastoma histotypes and lead to false negative exam, thus limiting the effectiveness of ^18^F-FDG-PET/CT in staging hepatoblastoma [30]. Besides, as described for other tumors, an acute hyperglycaemia at the time of the exam can compete with tracer uptake and lead to false negative reports [17]. Conditions leading to false positive ^18^F-FDG-PET/CT examinations include regenerating liver tissue and areas of inflammation (like necrotizing granulomas) and should be reminded when *α*-FP levels are only slightly or moderately elevated [15, 17]. 

Also, respiratory motion in children can be a problem causing misregistration of PET and CT scans and leading to attenuation correction artifacts, as in the case of a focal uptake in the dome of the liver, which could be falsely localized to the lung base on the attenuation-corrected images [31]. These pitfalls are due to the difference in diaphragm position at CT and PET. Misregistration may be minimized by performing CT during midexpiration [32–34]. Last but not least, radiation doses provided by additional imaging investigations to pediatric patients should be carefully evaluated as suggested by former papers [35, 36]. The effective dose provided by one PET/CT scan, according to our imaging protocol, ranges from 7.3 to 9.3 mSv, whereas the effective dose for an infant undergoing high quality abdominal CT is about 8–10 mSv (tube voltage = 120 kVp; tube current = 80 mAs; abdominal pitch of 1.5 : 1). 

## 5. Conclusions

PET/CT is nowadays an emerging diagnostic tool, which is acquiring an increasing importance in pediatrics. Our experience suggests that ^18^F-FDG-PET/CT scan may be used in children with HB without collateral effects and may provide incremental diagnostic value over CI in restaging and followup. Considering the importance of restaging disease after surgical approach, we stress the importance of rapid and correct disease extent definition in the event of recurrence. The association between morphological exams and functional images acquired at the same moment seems to be striking in the definition of disease extent. However, it is important to carefully evaluate, case by case, the necessity of performing PET/CT in hepatoblastoma in order to minimize the radiation exposure in pediatric patients. As this pathology is rare, more complex multicentre trials are warranted to suggest the introduction of ^18^F-FDG-PET/CT in the routine imaging workup for hepatoblastoma staging and in case of suspicion of relapse.

## Figures and Tables

**Table 1:** Characteristics of patients.

Patient number	Surgery	*α*-FP increase	Biopsy	Recurrence	CI findings	^ 18^F-FDG-PET/CT findings
1	Tx	Yes	Done	Yes	Hepatic recurrence	Additional abdominal findings
2	Tx	No	Done	Yes	Negative	Hepatic recurrence
3	Tx	Yes	Done	Yes	Hepatic recurrence and lung metastases	Additional lung lesions
4	Tx	Yes	Done	Yes	Hepatic recurrence	Additional hepatic lesions
5	Sx	Yes	Not done	No	Negative	Negative
6	Sx	Yes	Done	Yes	Negative	Epigastric lesion
7	Tx	Yes	Done	Yes	Negative	Diaphragmatic lesion
8	Sx	Yes	Done	Yes	Perisplenic lesion	Additional perisplenic lesion
9	Tx	Yes	Done	Yes	Negative	Abdominal wall lesions

CI: conventional imaging; Tx: liver transplantation; Sx: hepatectomy.

**Table 2:** Characteristics of all studies reporting patients with hepatoblastoma evaluated by PET or PET/CT.

Authors	Year	Journal	Country	Study design	Number of patients	Device used	Sex of patients	Mean age (years)	Indication
Sironi et al. [14]	2004	American Journal of Roentgenology	Italy	Case report	1	PET/CT	1 M	4	Restaging after Tx
Wong et al. [15]	2004	Journal of Pediatric Surgery	Hong Kong	Retrospective	16	PET	8 M; 8 F	2	Restaging after Sx
Figarola et al. [12]	2005	Pediatric Radiology	USA	Case report	1	PET/CT	1 M	3	Restaging after Sx
Philip et al. [16]	2005	Pediatric Surgery International	Australia	Case series	3	Coregistered PET and CT	NR	3	Restaging after Sx
Mody et al. [17]	2006	Pediatric Blood and Cancer	USA	Prospective	5	PET	3 M; 2 F	2	Restaging after Sx; response evaluation after Ch

M: male; F: female; Tx: liver transplantation; Sx: surgery; Ch: chemotherapy.
